# Selectivity and Internal Migration: A Study of Refugees' Dispersal Policy in Sweden

**DOI:** 10.3389/fsoc.2019.00066

**Published:** 2019-09-13

**Authors:** Yitchak Haberfeld, Debora Pricila Birgier, Christer Lundh, Erik Elldér

**Affiliations:** ^1^Department of Labor Studies, Tel Aviv University, Tel Aviv, Israel; ^2^Department of Economy and Society, University of Gothenburg, Gothenburg, Sweden

**Keywords:** refugees, dispersal policy, self-selection, economic assimilation, Sweden, internal migration

## Abstract

Following the intensified waves of refugees entering Europe, dispersal policies for newly arrived refugees have been proposed to speed up their integration and to share the financial burden across and within the EU countries. The effectiveness of dispersal policies depends, among other factors, on the extent to which refugees tend to stay in the initial location they are assigned to live in, and on their patterns of self-selectivity during subsequent moves of internal migration. Economic theories of migration suggest that economic immigrants are self-selected to destinations based on their abilities. Highly skilled and motivated people tend to migrate to labor markets with broader opportunity structures, while less capable individuals choose markets that are more sheltered. We use a quasi-experimental design to examine the extent to which those theories are first, applicable to refugees as well, and second, explain their self-sorting into local labor markets at destination. We focus on a refugee cohort that came to Sweden during the period when the so-called “*Whole-Sweden*” policy was in effect. This policy was designed to reduce the concentration of refugees in the larger cities by randomly deploying asylum seekers across Sweden. After being assigned to an initial location, refugees could move freely within Sweden. We use individual register data from Statistics Sweden to study all refugees who arrived in Sweden during 1990–1993, and we follow each one of them during an 8-year period. We use discrete-time survival analysis (complementary log-log models) in order to assess the effects of abilities on the destination choices of refugees, and individual fixed-effect models to assess the effects of internal migration on their income. Destinations were defined on the basis of the economic opportunities they offer. The results suggest that refugees' education levels are related to major differences in their destination choices. Highly skilled refugees were more likely to migrate to labor markets with a wide structure of opportunities relative to less skilled refugees. In addition, all relocation choices had positive effects on refugees' income growth.

## Introduction

Economic theories of migration suggest that economic immigrants are self-selected to destinations based on their abilities. Highly skilled and motivated people tend to migrate to labor markets with broader opportunity structures, while less capable individuals choose markets that are more sheltered. The purpose of this study is to assess the extent to which theories explaining the behaviors of international economic migrants and their economic assimilation at their destination countries are first, applicable to refugees as well and second, relevant in the context of their self-sorting into local labor markets at destination.

Specifically, we ask two questions that are relevant to the ongoing debate about the effectiveness of dispersal policies of refugees, mainly in Europe. First, are refugees self-sorted into different labor markets within their host country based on their skills and abilities? Second, whether those self-sorting patterns are economically rewarding to the refugees.

For that, we focus on a refugee cohort that came to Sweden during the period when the so-called “*Whole-Sweden*” policy was in effect. This policy was designed to reduce the concentration of refugees in the larger cities by randomly deploying asylum seekers across Sweden. After being assigned to an initial location, refugees could move freely within Sweden. We follow each one of them in order to assess first, the effects of their abilities on their destination choices and second, the effects of their internal migration patterns on their income.

### Migrants' Dispersal Policies

Following the increase in the number of refugees coming to Europe a few years ago, several mechanisms for distributing asylum seekers across and within European countries have been debated. Advocates of such policies argue that allocation policies can help in sharing the burden of absorption of refugees across the EU and in facilitating their integration. Similarly, some countries favor this approach when implementing their own integration policies as a mean of reducing the financial and social burdens of immigration that fall upon refugees' preferred destinations—usually the countries' major cities. However, two key questions remain unanswered, namely, (1) to what extent refugees allocated to different areas of the host country stay in their initial locations, and (2) whether such policies contribute to the integration of immigrants.

There are several examples of countries that have implemented dispersal policies for asylum seekers in the past. Research on the effectiveness and consequences of such policies has yielded contradictory results. A study conducted in Scotland suggested that most individuals remained in their assigned sites, and questioned the impact of constrained mobility on refugees' opportunities for social and economic integration (Stewart, 2012). In Denmark, negative selectivity of refugees' relocations after being randomly settled across the country was found. Immigrants with low skill levels were more likely to move to ethnic enclaves in major cities. However, these low-skilled movers showed a significant increase in their earnings following their change of residence (Damm, 2009). These findings suggest that ethnic networks are important for matching individuals with jobs. Similarly, results from the Netherlands indicate that there are important economic benefits for immigrants who reside in neighborhoods where there is a high concentration of members of their own ethnic group, when other neighborhoods' characteristics are controlled for (Beckers and Borghans, 2011). Several studies have examined the effectiveness of the Swedish settlement policy and found that it had negative impacts on the economic integration of immigrants. One explanation put forward by Edin et al. (2004) is that the policy shifted the emphasis from labor market integration of refugees to their income support by the state. It was also found that refugees tend to move to larger cities where members of their ethnic group are present and where there are more employment opportunities. However, no increase was found in the intensity of secondary migration of refugees that were part of the program relative to those that were not affected by it (Andersson, 1998; Åslund, 2000, 2005). Given the inconsistent results of previous research, we suspect that the effectiveness of the policy depends on the motives, and consequently on the selectivity patterns of individuals in staying or moving out of their assigned locations.

### Theoretical Framework

The economic theory of international economic migration suggests that patterns of immigrants' self-selection influence immigrants' economic performance at their destinations. Obviously, receiving countries' characteristics serve as signals for prospective immigrants that choose among destinations, and at the same time—affect their assimilation after arriving there. Most research on the impact of the interaction between (1) immigrants' self-selection patterns from their countries of origin; (2) host country characteristics; and consequently (3) immigrants' sorting into those destinations—on the economic assimilation of immigrants, has been centered on migration waves across countries. We try to adopt these theories in our attempt to explain refugees' sorting into local markets in one country (Sweden), and their earnings assimilation in their chosen local markets there.

### Self-Selection, Sorting, and Assimilation

Scholars of international economic migration have pointed at two main interrelated determinants of immigrants' economic assimilation namely, immigrants' patterns of self-selection from their countries of origin (Chiswick, 1978; Borjas, 1987), and the host country's reception context (Borjas, 1990; Portes and Rumbaut, 2006). Understanding the joint contribution of these two factors on the economic assimilation of immigrants has important implications for policy-making (Borjas, 1990).

Immigrants' patterns of self-selection are one of the main determinants of their economic assimilation. The concept of “self-selection” was originated by Roy (1951) in the context of occupational choice, but has since been applied to many types of rational choice-making. Chiswick (1978) introduced it in the study of decisions made by potential immigrants at source countries whether, and where to migrate. He, and other scholars, argued that immigrants are not a random sample drawn from the source country population, but rather represent a positively self-selected group from the population at risk. Migration entails risks and costs that immigrants decide to take in order to improve their economic conditions at the destination country (Chiswick, 1979; Borjas, 1987; Chiswick and Miller, 2005).

Immigrants choose to migrate to destinations where the demand and consequently the relative compensation for their skills is the highest. The characteristics of potential destinations can, therefore, be evaluated by various measures of inequality that reflect differences in the relative remuneration by labor markets to varying levels of qualifications. That is, individuals with high levels of observed and unobserved qualifications tend to migrate to places where there is a high level of inequality, because there they receive higher returns to their skills relative to low-inequality destinations. In contrast, individuals with low skill levels tend to migrate to markets where there are low levels of inequality, since the “penalty” that accrues to their low skills is relatively smaller, and their relative position along the income distribution is closer to the mean.

Furthermore, when immigrants have several destinations to choose from, then additional sets of within-immigrants sorting patterns play a role in determining the distribution across destinations of those who decide to migrate. For example, Grogger and Hanson (2011) found that the gaps in education between immigrants and non-migrants from their own source country get wider (favoring the immigrants), as the skill-related difference in earnings between the destination and source countries gets larger. Descriptive statistics indeed confirm that immigrants are generally positively self-selected from the population at risk namely, they are more educated than their non-migrant counterparts at their countries of origin (Docquier and Marfouk, 2006).

Some studies that have examined self-selection patterns involved in internal migration assumed that individuals are randomly distributed in different regions (Borjas et al., 1992; Gabriel and Schmitz, 1995; Nakosteen et al., 2008; Abramitzky, 2009). For example, Borjas et al. (1992) examined the self-selection of individuals within the United States during the 1970s, assuming that there is no correlation between individual characteristics at the age of 14 and the average level of compensation where they reside. Clearly, such an assumption is questionable, because it assumes that there was no self-selection in the parents' generation to regions with high rewards to their skills, nor any intergenerational transfer of skills between parents and their children^1^. Yet, some results suggest that immigrants are self-selected and that inter-state differences in returns to skills are a major determinant of both the size and skill composition of internal migration flows in the U.S. That is, immigrants are self-selected based on the differences in the returns to their skills in their state of origin and the other states they migrate to e.g., Gabriel and Schmitz (1995).

### Context of Reception and Assimilation

The second main determinant of immigrants' economic assimilation is the destination's characteristics, including migration and welfare policies, and market structure. Clearly, the destination's reception contexts affect the type of immigrants that prefer to arrive to certain locations and consequently, their patterns of sorting into those places.

In the context of within-countries migration, the more relevant explanation is that referring to the nature of local rather than national labor markets. It suggests that the economy is divided into primary (where the demand is for highly skilled workers, with highly paid jobs and career opportunities) and secondary (with low-skilled, low-paying jobs) labor markets. The primary labor market is characterized by a broader structure of opportunities relative to the secondary labor market. Individuals with higher qualifications therefore tend to self-sort themselves to primary labor markets, while the entry of individuals with low levels of qualifications to these markets is restricted. Consequently, low-skilled workers are expected to have better employment opportunities in secondary labor markets (Piore, 1970, 1979). The labor market's structure of opportunities is operationalized in the present study by three variables: the size of the labor market, mean earnings in the market, and the percentage of those who have an academic degree.

Economic theories therefore suggest that individuals tend to migrate to where they expect to receive the highest returns to their skills and abilities. Since there are differences in the returns to skills across regions and localities within countries, it can be hypothesized that internal migration should not be different from international migration. Highly skilled individuals are expected to migrate to regions where there are high returns to their skills, while the less-skilled ones tend to stay in or migrate to regions where their loss due to their lack of skills is smaller (Borjas, 1987, 1990). Individuals that suffer from the highest levels of mismatch between their characteristics and the structure of returns in their region of residence are the most likely to internally move. Therefore, the differences in pay-to-skills across regions affect the skills distribution of internal migration flows (Borjas et al., 1992). Additionally, some studies have attempted to also identify self-selection patterns of immigrants based on unobserved characteristics that are assumed to also affect economic assimilation (e.g., Borjas, 1990; Saarela and Rooth, 2006; Cohen and Haberfeld, 2007; Haberfeld, 2013). Examples of unobserved attributes include risk aversion, motivation and other individual characteristics that have major implications for immigrants' labor market outcomes.

### Economic Assimilation of Immigrants

As described above, immigrants' self-selection and sorting patterns comprise of both observed (mainly measured by their education level) and unobserved characteristics (such as motivation and risk-taking). A positive self-selection pattern on both observed and unobserved attributes enhances migrants' ability to economically assimilate in the host country (e.g., Borjas, 1990; Cohen and Haberfeld, 2007; Haberfeld, 2013).

Students of international migration have suggested that immigrants (regardless of their specific levels of human capital) experience considerable social and economic hardships in the labor market of the host society upon arrival (e.g., DeVoretz, 2006). Immigrants at that stage are not familiar with the new labor market; they have limited access to information and to social ties; they do not have full command of the language; their occupational skills are not always fully transferable to the new economic system, and at times they even face discrimination. As a result, immigrants (even high-skilled) are at a disadvantage upon their arrival when compared to native-born workers of similar attributes (Chiswick and Miller, 2009).

With the passage of time in the host society, however, many immigrants experience upward occupational and economic mobility, and consequently, improve their relative market position. Indeed, after a certain period of time in the host society immigrants have been found, many times, to close the earnings gaps with comparable native-born populations, especially among those with high levels of human capital (Chiswick, 1978, 1979; Borjas, 1990; LaLonde and Topel, 1997).

Although human-capital is highly influential in shaping immigrants' economic fortunes, the context of reception prevalent in a specific market mediates the effect of training and skills (and specific occupations) on the incorporation of highly skilled immigrants into that market. Research on international migration in several countries also suggests that economic assimilation of highly skilled immigrants may not be taken for granted and depends on countries' migration policies, citizenship laws, economic opportunities in the labor market, the occupational labor market in which the immigrant worker operates, and welfare institutions—among others (Cohen and Haberfeld, 2007; Chiswick and Miller, 2009).

Obviously, internal and international migrations are very different on key issues. For example, the issue of immigrants' citizenship of the host country is much more important for assimilation at the international than the internal migration processes. Yet, we can derive important insights from the international migration theory and research literature for better understanding movements from one locality to another within the same country. Issues such as market structure, occupational markets and their barriers faced by newcomers, or welfare policies are relevant in local as much as in national markets.

### Theoretical Shortcomings

While our main conceptual model is driven from an economic perspective, the literature offers non-economic explanations as well for location choices by individuals. Aradhya et al. (2017) argue that immigrants' location decisions should be understood as part of a broad utility model in which residential choices of immigrants are the result of a wide range of their residential preferences, both economic and non-economic. Most importantly, studies have suggested that a major motive for immigrants' choice of destination concerns being closer to relatives, friends, and co-ethnic group members (e.g., Massey et al., 1993; McPherson et al., 2001; Epstein, 2002; Bauer et al., 2007; Aradhya et al., 2017).

When individuals live in proximity to their relatives, friends, and co-ethnic group members, they will move to other places only for total gains that exceed the direct costs of moving along with the emotional costs of leaving their social environment (Dahl and Sorenson, 2010). Similarly, Chiswick and Miller (2005) hypothesized that migrants are willing to accept lower wages if the job offered to them is located in an ethnic enclave, because of the non-economic benefits labeled as “ethnic goods.” Dahl and Sorenson (2010) developed a new methodology for determining how individuals weigh both financial and social factors in order to predict geographic mobility. They show that when immigrants take internal migration decisions, their preferences for living near relatives and friends are more important than opportunities for higher pay elsewhere. Consequently, if individuals are initially randomly placed, then some of them might decide to relocate themselves in order to be closer to relatives or other members of their ethnic group, despite a possible economic loss associated with such a relocation decision.

Available housing alternatives are another key factor in immigrants' (and probably even more so in refugees') choices of destination. In most cases, housing is the costliest item in immigrants' expenses. Consequently, it is reasonable to assume that when considering geographical relocation, immigrants (including refugees) assign a high weight to the cost of housing at both their present location and at relocation alternatives. It is quite possible that relocation decisions made by refugees following their initial residential placement are affected by housing considerations and not necessarily by earnings, social, or environmental motives.

Finally, some scholars have lately noted that environmental motives increasingly compete with the abovementioned economic and social reasons for interregional migration (Lundholm, 2007; Bonasia and Napolitano, 2012; Vilhelmson and Thulin, 2016). Environmental motives could include the specific qualities of a potential destination, such as the natural setting of the place or its social and cultural environment^2^. Clearly, the studies that emphasize environmental motives are more relevant to non-refugees (when many destinations can be easily chosen).

That being said, the ability to empirically distinguish between first, economic and non-economic, and second, between various non-economic motives for migration is many times limited. Furthermore, there are non-economic motives for migration that might affect migration's economic outcomes in non-trivial ways. For example, preferences for living close to relatives, friends and co-ethnic group members clearly, enhance immigrants' networks that reduce the cost of migration and facilitate the migrants' economic integration in their new locations. Not only that, self-sorting patterns could be related to different types of immigrants' networks. It is quite possible that high- and low-skill immigrants join different networks across and within geographical locations. Furthermore, different networks might help immigrants in attaining different economic outcomes (for example, employment opportunities vs. high earnings). Similarly, lowering housing costs can be viewed as an economic motive, but it is not related necessarily to immigrants' motive to raise their earnings levels.

Here, we follow the economic framework of immigrants' self-selection (Chiswick, 1978; Borjas, 1987, 1990), deriving from it our expectations as well as our empirical approach for analyzing refugees' internal moves within Sweden. Notwithstanding, we link this framework to the immigrants' networks explanation due to the close linkage between the two.

## The Setting

Until the beginning of the 1980s, immigration to Sweden was composed mainly of labor migrants from Nordic, West, and South European countries. Therefore, immigration was mainly seen as an economic issue related to the operation of the labor market and hence was handled by the Labor Market Board (AMS). The Board handled issues of immigrants' recruitment, their integration in Sweden, and the assignment of residence location to quota refugees. However, since the mid-1970s, immigration to Sweden has contained increasing numbers of refugees from a broad range of countries and cultures (Bengtsson et al., 2005). As a result, in 1985 the Immigration Board (SIV) replaced AMS in managing international migration and a new dispersal policy was introduced.

This new policy became known as the “Sweden-wide strategy” or “The Whole-of-Sweden Strategy” (Andersson, 1998; Bevelander, 2010). The reform was introduced in response to complaints from cities that had experienced a rise in the number of incoming immigrants and perceived this as a burden on their local resources. SIV was given the authority to assign newly arrived refugees to their initial municipality of residence. By implementing this policy, the government hoped to speed up the integration process of refugees and to reduce the burden on the public budget of the big cities (Åslund et al., 2011). Therefore, arriving asylum seekers could not freely choose their place of residence. Rather, agreements were signed between SIV and the municipalities on the number and types of refugees (ethnic/linguistic origin, families/singles) that would be assigned to each municipality (Andersson, 2007). The municipalities received from the government budgets to cover the cost of housing and board for asylum seekers and refugees during their first three years in the assigned localities, and a fixed sum of money for other costs (RegSkr, 1986/1987:134, p. 27).

Originally, municipalities were selected based on their ability to incorporate immigrants. However, over time the number of receiving municipalities increased from 60 to 277 out of Sweden's 284 municipalities in 1989 (Åslund et al., 2011), including also some municipalities that experienced a net out-migration flow because of a depressed local economy (Bevelander, 2010). As the number of refugees rose over the years, public housing availability determined immigrants' placement rather than effective local integration policies (Åslund et al., 2011).

It should be noted that while refugees were given little choice in their initial placement, they were free to move if they were able to find housing in other municipalities. Although the only direct cost for refugees as a result of moving was losing their place in language courses (i.e., delaying their enrolment; Åslund et al., 2011), the refugees' ability to find housing by themselves was extremely limited due to the tight housing market in Sweden during that period. The policy was abandoned in July 1994 as a result of the long waiting time in refugees' reception camps for apartments in the participating municipalities. Since 1994, asylum seekers have been able to look for their own housing and are entitled to housing allowances, or alternatively a free place in an asylum location (RegSkr, 1994/1995:131, p. 29).

## Expectations

As stated, our conceptual model is driven by an economic perspective; therefore our expectations are basically in line with prevalent economic theory. We hypothesize that refugees that are assigned to their initial location, similarly to economic immigrants, conduct an evaluation of their economic position (i.e., employment possibilities and earnings) in their initial placement and their expected position in the labor markets they consider relocating themselves to. Consequently, we hypothesize that refugees with higher education levels move to (or stay in) labor markets with broader opportunities structures (as captured empirically by the size of the labor market, mean earnings in the market, and the percentage of those who have an academic degree), and that lower-educated refugees select labor markets with narrower opportunities structures. *In both cases*, refugees are expected to gain economically from their relocations.

However, a main caveat might change these basic economic predictions in different ways. The Swedish case is in many respects unique and different from situations studied in previous migration research. First, during the period in which the refugees arrived, Sweden suffered a major economic crisis that could limit internal migration among refugees. Following that crisis, the country has been experiencing structural transformation since the early 1990s (Åslund and Rooth, 2007). Second, Sweden is still (as compared to most other countries) a leading welfare state. Therefore, the incentives and possibilities faced by refugees in Sweden who seek to relocate could be more extensive than simply employment opportunities and higher earnings—as hypothesized in other countries.

## Data

We use Swedish Register data from GILDA^3^, which cover the entire Swedish population from 1990 to 2014. The register contains longitudinal information on place of birth, immigration year, income from salary, age, education, and place of residency. We study immigrants that arrived in Sweden between 1990 and 1993 at the age of 25 to 55 upon arrival, and follow each of them for 8 years since their year of arrival: for example, we follow those who arrived in 1992 for each year until 1999. Unfortunately, the data do not contain information about immigrants' visa type (at least not for these years). Therefore, we focus on immigrants from nine leading source countries: Yugoslavia^4^, Bosnia-Herzegovina, Croatia, Slovenia, Macedonia, Somalia, Lebanon, Iraq, and Iran. Immigrants from these source countries can be viewed as refugees since most of them came to Sweden as asylum seekers and were therefore part of the settlement program^5^. In total, full information on all variables for those 8 years (the year of their arrival plus the seven subsequent years) for 31,508 individual refugees is available and was included in the analysis.

## Methods

There are two main analyses. First, we examine whether the tendency of refugees to relocate to (or stay in) different labor markets is related to their levels of education. In the second stage, we examine the impact of those relocations on income growth after migration. We treat a movement from one labor market to another as our unit of analysis. Since municipalities cannot serve as an accurate measure of labor market opportunities, we use Statistic Sweden's definition^6^ of local labor markets, which includes 112 local labor markets in Sweden. In order to do so, we harmonized the labor markets to the 1990 definition^7^.

### Movement Models—Selectivity in Internal Migration

In the first stage, moving between two labor markets that differ in their structures of opportunities is the dependent variable (did not move or moved to a similar-opportunities market; moved to a wider-opportunities market; moved to a narrower-opportunities market). Following findings by Bevelander and Lundh (2007) and Hedberg and Tammaru (2010) on the factors affecting the economic success of refugees in Sweden, the labor market's structure of opportunities is calculated as an index of three variables: size of the labor market (number of people living there), mean earnings in the market, and the percentage of those who have a BA (or a higher degree). We re-calculated the index for each one of the 11 years examined in this study (1990–2000). Each of the variables included in the index was then standardized to a 1–100 scale. The final index is the mean of these three standardized variables (with a mean of 84.3, and s.d. of 16.1). The inter-item reliability coefficient of the opportunities structure index ranges from 0.63 to 0.88 during 1990–2000.

In this stage of the analyses, we use discrete-time survival analysis [complementary log-log (c-log-log) models] to examine the internal migration patterns of refugees during their first 8 years in Sweden. We assess whether, in line with our expectations derived from the migrants' self-selection logic, highly skilled refugees were more likely to migrate to markets with broader structures of opportunities and low-skilled immigrants to more sheltered labor markets, as opposed to remaining in their initially assigned markets. The omitted category in this model is staying in the assigned labor market (or internal migration to a different labor market that has the same level of opportunities structure). We examine the risks of two events. The first is internal migration to a market with a broader structure of opportunities (defined as a difference of more than 10 units between the two markets on the scale of 1–100 of the index, which equals to more than 0.6 s.d. of the index). The second event is internal migration to a more sheltered labor market (again, more than a 10-unit difference between the two markets on the index).

### Income Models—the Economic Consequences of Internal Migration

In the second stage of the analyses, we checked the consequences of the internal move (or lack of) to the main labor-market outcome—earnings. We estimate the impact of the above migration decisions on refugees' income growth, using individual fixed-effect models. These models follow a method offered by Bratsberg and Raaum (2011) to assess the effect of citizenship acquisition on earning (see also Helgertz et al., 2014). The model is represented by the following equation:

$$\begin{array}{lll} ln\left(y_{it}\right) & = & a_{0}M_{it}+a_{1}M_{it}\left(X_{it}-X_{iM}\right)+a_{2}D_{i}X_{it}+{\gamma \text{X}}_{it}\\ \; & + & \delta {\text{Z}}_{it}+\varepsilon_{i}+u_{it} \end{array}$$

The dependent variable—ln(y_it_)—is measured as the natural logarithm of an individual's (i) labor earnings in a given year (t)^8^ (adjusted by using the Consumer Price Index—KPI).

We focus on four main parameters:

1. **X**_it_ represents the individual's time at destination (i.e., Years Since Migration—YSM), measured as a sequence of yearly dummy variables, and ****γ**** is the estimated coefficients vector of refugees' annual *assimilation rates in Sweden*. Based on the standard immigrants' assimilation model (e.g., Chiswick, 1978), immigrants are expected to experience earnings growth above and beyond that of natives of similar attributes, particularly during their first years at the host country, regardless whether they move within that country or not.
2. In order to test if those refugees who chose to move within Sweden are *positively self-selected*, a separate effect from YSM is estimated for those who, at some point during the time of observations, move. This is the effect (a_2_) of the interaction between YSM and a dummy variable (Di) that indicates whether the individual moves at some point. It indicates whether those who move enjoyed a higher assimilation rate already *prior to their move* compared to those who chose not to move.
3. The ***M***_*it*_ are two time-varying dummy variables—one for a move to a market with a wider structure of opportunities, and the other for a move to a narrower structure—with the value “1” for individuals who moved within Sweden after their initial placement and the value “0” otherwise. It is designed to test our expectation that movers improve their economic standing—as predicted by the economic framework of migration (whether international or internal). The first movement (of both types) is the only one to be considered^9^. These two variables capture whether the effect of moving within Sweden is a *one-time shift in earnings* (i.e., an earnings premium). Therefore *a*_0_ represents a shift effect of moving, which is assumed to be constant in all years following the internal migration.
4. We also allow for a *differential earnings slope after internal migration*, estimated by the parameter *a*_1_, using a variable measuring the effect of years since internal migration. This parameter is derived from a continuous variable, constructed as *X*_*it*_ – *X*_*iM*_, representing the difference between the individual's time in Sweden (YSM = (*X*_*it*_)) and the number of years since internal migration (*X*_*iM*_). A positive *a*_1_ indicates an earnings growth subsequent to the move that is steeper above and beyond the two shift coefficients (that of YSM (*X*_*it*_) – ****γ****, *and that of M*_*it*_− *a*_0_), whereas a negative *a*_1_ coefficient implies that the yearly earnings growth prior to the move is greater than after it.

**Z**_*it*_ is a vector of time-varying control variables including education, marital status, and lag-labor market characteristics. Finally, ε_*i*_ represents time-constant unobserved characteristics at the individual level, as estimated by using fixed-effect OLS regression, and *u*_*it*_ is the time-varying unobservable variance.

Clearly, our model cannot separate all variables that lead to earnings growth of stayers and movers—other than the above estimated four parameters and those parameters associated with the control variables (**Z**_*it*_) described here. For example, it is quite possible that earnings growth of some movers that chose to change their localities due to social rather than economic motives resulted from newly established networks in the localities they move to, and not necessarily because of better earnings offers there. However, this estimation problem is not crucial if we assume that our fixed-effect model controls for time-invariant unmeasured attributes such as preferences for living in localities with certain non-economic characteristics.

## Variables

The estimated models include the following variables:

*Education*–A sequence of four dummy variables for the highest-level education completed (elementary^10^, secondary, post-secondary non-academic, and academic) in which the omitted category is completing secondary education. This is the one focal variable in the first stage of the analyses, as we are interested in evaluating whether refugees with higher abilities and skills (as captured by their education) are more prone to moving within Sweden.

*Year*–The population includes immigrants arriving during 1990–1993, followed for eight subsequent years starting at their year of arrival. In the first stage of the analysis, we include a control for the year of the observation (year = 1990, 2000)^11^.

*Age*–Immigrant's age on December 31 of each year. As we restricted the age at migration to 25–55, the age of refugees ranges from 25 to 62. In the first stage of the analysis, we also include indicators for *gender* (female = 1), *marital status* (married = 1), *lag employment status* (employed = 1), and *country of origin* (the omitted country is Yugoslavia). In the second stage, we divided the sample by country of birth and gender in order to be able to control for time-invariant variables in the fixed-effect models, and control for marital status with a dummy.

Finally, we incorporate labor market-level variables in the individual-level models, in order to control for market-level variables that might affect people's decision whether to relocate^12^.

*Lag labor market unemployment level–*This variable represents the mean unemployment days for which unemployment benefits have been paid in the labor market 1 year prior to the move (at t-1) (calculated as the total days of unemployment in the labor market divided by the number of individuals in the labor market). This variable serves as a proxy for the employment levels in the labor market in which immigrants live before deciding whether or not to move.

*Lag immigrant-groups representation in the labor market*–This variable indicates the concentration level of each one of the ethnic groups studied in the labor market 1 year prior to the immigrant's move out of it (at year t-1). It is (the natural logarithm of) the ratio of the percentage of each ethnic group in the specific labor market divided by the percentage of the same ethnic group in the entire Swedish population. The variable ranges from −5 to +5, where a positive number indicates an overrepresentation of the ethnic group in the local labor market (i.e., the proportion of the specific ethnic group is higher than their general share of the population), and a negative number an underrepresentation in that labor market.

In the first stage, we also control for *LAG opportunity index level*. This controls for the opportunity level in the first place in which individuals reside.

## Results

### Descriptive Statistics

Table 1A presents the descriptive statistics of the individuals included in the refugee population, and Table 1B presents the same descriptive statistics by type of internal migration in Sweden (averages at the individual level for all years).

**Table 1A:** Descriptive statistics of all refugees 1 year after they immigrated to Sweden between 1990 and 1993, at the ages of 25–55, their labor market characteristics, and their migration decision.

**Variable**	**Mean *(SD—between)***
Age	37.64
	*7.19*
Female	0.44
Married	0.63
YSM	3.44
	*0.62*
**Education level**	
Elementary	25.94
Secondary	45.11
Post-secondary non-academic	15.95
BA+	25.67
**Birthplace**	
Yugoslavia	14.61
Croatia	1.02
Slovenia	0.10
Bosnia-Herzegovina	32.83
Macedonia	0.55
Somalia	5.86
Lebanon	8.13
Iraq	18.12
Iran	18.78
**Migration year**	
1990	15.63
1991	16.31
1992	15.82
1993	52.25
**Labor market variables**	
Lag Mean LM unemployment days	22.30
	*3.14*
Lag immigrants representation	0.17
	*0.53*
LM structures of opportunities	84.33
	*16.09*
**Internal migration structure of opportunities**	
Stay	64.97
Moved to wider structure of opportunity	27.39
Moved to narrower structure of opportunity	7.64
Employed	0.31
Ln earnings (*Individuals with positive earnings only)	10.75
	*1.22*
*N of individuals-All*	*31,506*
N of observation- All (individual * year)	*237,708*
*N of individuals- with positive earnings*	*23,585*
N of observation-with positive earnings (individual * year)	*85,585*

**Table 1B:** Descriptive statistics of the refugees and their labor markets 1 year after migration to Sweden by internal migration type, refugees that migrated to Sweden between 1990 and 1993, at the ages of 25–55.

**Variable**	**Stayed**	**Moved to a wider structure**	**Moved to a narrower structure**
Age	37.91	37.32	36.27
	*7.29*	*6.96*	*6.87*
Female	0.45	0.42	0.40
Married	0.62	0.63	0.64
**Education level**			
Elementary	26.04	26.1	24.51
Secondary	44.14	46.31	49.11
Post-secondary non-academic	16.32	15.56	14.25
BA+	26.61	24.34	22.52
**Birthplace**			
Yugoslavia	16.12	10.62	16.04
Croatia	1.11	0.86	0.83
Slovenia	0.13	0.05	0.08
Bosnia-Herzegovina	29.8	37.41	42.09
Macedonia	0.64	0.35	0.46
Somalia	4.58	8.68	6.65
Lebanon	8.51	7.33	7.73
Iraq	19.44	16.34	13.38
Iran	19.67	18.36	12.75
**Immigration year**			
1990	15.38	17.05	12.59
1991	15.67	18.04	15.54
1992	17.86	12.07	11.88
1993	51.09	52.83	59.99
**Labor market variables at the first year after migration (YSM** **=** **0)**			
Mean LM unemployment days	21.46	23.22	23.21
	*6.46*	*7.45*	*6.74*
(Ln) immigrants representation	0.15	0.19	0.00
	*0.60*	*0.53*	*0.67*
LM structures of opportunities	87.25	57.43	85.94
	*15.80*	*21.54*	*13.02*
**Labor market outcome at first year (YSM** **=** **0)**			
Employed	0.07	0.03	0.03
(Ln) income from work and self-employment	9.76	9.06	9.41
	*1.46*	*1.58*	*1.55*
**Labor market outcome after 7 years (YSM** **=** **7)**			
Employed	0.56	0.53	0.66
(Ln) income from work and self-employment	11.34	11.27	11.51
	*1.29*	*1.32*	*1.26*
*N of individuals-All*	*20,468*	*8,631*	*2,407*
N of observation- All (individual * year)	*158,174*	*63,924*	*15,610*
*N of individuals- with positive earnings*	*15,687*	*6,196*	*1,702*
N of observation-with positive earnings (individual * year)	*59,537*	*20,314*	*5,734*

Starting with Table 1A, it can be seen that the mean age of immigrant refugees is 37.6 years, and more than half of them are men and married. Most refugees have completed secondary education and over 25 percent have higher education. About half of this cohort of refugees is composed of refugees from war zones in the Balkans (mainly due to the Yugoslav War, also termed the Third Balkan War). About 33 percent of them are from Bosnia-Herzegovina, and almost 15 percent of them from other regions of the former Yugoslavia. The second half of this cohort arrived almost entirely from the Middle East. Among them, the two largest groups, amounting to 18 percent each, are from Iraq and Iran, while a smaller group originated in Lebanon (about 8 percent). Finally, <6 percent come from Somalia. Most of the refugees belonging to the 1990–1993 cohort arrived in 1993 as a result of the escalation of the Third Balkan War.

There are three main labor market variables we are interested in: the index of labor market opportunities structure, average days of unemployment, and own ethnic group representation. Most importantly, the mean index of labor market opportunities is 84.33 with a standard deviation of 16.9. The average paid days of unemployment across labor markets is 22, and the average level of immigrants' representation in the labor market in all years is 0.17, which indicates a small overrepresentation of immigrants in the markets in which they were settled.

Finally, turning the focus to internal migration patterns, ~27 percent of the refugees moved to labor markets with a wider structure of opportunities, and 7.6 percent moved to narrower-structure markets. The remaining two-thirds of this refugee cohort chose to stay in their assigned locations (or to move to another, but with similar opportunities level).

Table 1B presents the same descriptive statistics presented in Table 1A, but separately for the type of internal migration based on labor market structure of opportunities. In general, we can see that refugees moving to other destinations tend to be men and somewhat younger than stayers. It also seems that those who stay in their assigned locations have higher levels of education (academic and post-secondary non-academic). The ethnic distribution of those who move differs from that of the general population (as can be seen from Table 1A). For example, larger proportions of refugees from the former Yugoslavia constitute the stayers in the labor markets where they were placed, as well as refugees from Iran and Iraq.

The middle part of Table 1B presents the characteristics of the labor markets in which the refugees were settled in their first year in Sweden. Comparing the labor market characteristics of immigrants that stay to those that move away from the labor markets in which they were placed right after migrating to Sweden allows us to examine whether those refugees who chose to relocate were placed in labor markets with distinct characteristics. As can be seen, the opportunities index of the assigned labor markets is lower in the markets in which the movers were settled, and even more so in the assigned markets of those who moved to labor markets with more opportunities^13^. In addition, the ethnic representation levels of the labor markets of individuals that eventually moved to narrower structures of opportunities are lower than those of the two others^14^. Finally, the immigrants that eventually decided to move were initially assigned to labor markets with higher levels of unemployment.

The bottom part of Table 1B presents the labor market outcomes of individuals in their first year in Sweden and the same outcomes seven years later—after their final decision whether and where to migrate. At the individual level, it is clear that refugees who eventually decided to move from their initial assigned location have lower rates of employment and lower income from work in their first year in Sweden. Overall, as expected, the employment rates of refugees shortly after immigration are very low (<10 percent), but the employment rates of those who decide to move are even lower. An examination of the refugees' labor market outcomes 7 years after migration provides an interesting picture. While at the beginning of the period refugees moving to labor markets with narrower structures of opportunities show low levels of employment, after 7 years this group has the highest levels of employment (66 percent of them are employed), as compared to 56 percent of refugees that stayed in their initial locations and 53 percent of refugees that moved to markets with wider structures of opportunities.

### Selectivity in Internal Migration

Table 2 presents the results of a discrete-time survival analysis [complementary log-log (c-log-log) models] examining the internal-migration patterns of refugees to a different labor market, based on different structures of opportunities, within the 8 years starting at their arrival in Sweden. The first model describes a movement of refugees from their initial assigned location to a location with a wider structure of opportunities. Similarly, the second model presents a movement to a labor market with a narrower structure of opportunities. Our main variable of interest is immigrants' education level. As can be seen from the first model, refugees with higher education levels (academic degree) are, as expected, more likely to move to labor markets with a wider structure of opportunities relative to individuals with secondary education, while their cohort fellows with lower education (elementary) do so less—when individual and labor market characteristics are controlled for. The second model shows that refugees with higher education (academic and post-secondary non-academic) are less likely to move to labor markets with a narrower opportunities structure relative to those with secondary education, while elementary-education refugees do so more^15^.

**Table 2:** Complementary log-log models of moving to labor markets with different structures of opportunities within 7 years in Sweden.

**Variables**	**Moved to a wider**	**Moved to a narrower**
YSM	0.881^***^	0.867^***^
	(0.015)	(0.025)
Age	0.992^***^	0.972^***^
	(0.002)	(0.003)
Female	0.958^*^	0.762^***^
	(0.024)	(0.032)
Year	0.873^***^	0.897^***^
	(0.014)	(0.023)
Elementary	0.910^***^	1.039
	(0.030)	(0.057)
Post-secondary non-academic	1.023	0.839^***^
	(0.040)	(0.055)
BA+	1.166^***^	0.912
	(0.041)	(0.053)
Marred	0.889^***^	0.883^***^
	(0.023)	(0.038)
Croatia	1.294^**^	0.765
	(0.168)	(0.177)
Slovenia	0.912	0.805
	(0.433)	(0.586)
Bosnia-Herzegovina	1.525^***^	1.515^***^
	(0.067)	(0.100)
Macedonia	0.899	0.702
	(0.182)	(0.217)
Somalia	2.212^***^	1.000
	(0.128)	(0.094)
Lebanon	0.987	0.748^***^
	(0.060)	(0.070)
Iraq	1.339^***^	0.577^***^
	(0.064)	(0.044)
Iran	1.328^***^	0.547^***^
	(0.065)	(0.043)
LAG employment	0.634^***^	0.728^***^
	(0.021)	(0.039)
LAG mean LM unemployment days	1.013^***^	1.028^***^
	(0.002)	(0.005)
LAG opportunity index level	0.954^***^	1.016^***^
	(0.000)	(0.001)
Lag Ln Immigrants Representation	0.850^***^	0.730^***^
	(0.014)	(0.023)
Constant	4.268^***^	0.025^***^
	(0.399)	(0.005)
Observations (individual ^*^ year)	*164,439*	*164,439*
*Individuals*	*31,450*	*31,450*
N_s	8,629	2,407
Ll	−25,375	−11,820

Robust s.e in parentheses.

*** p < 0.01,

** p < 0.05,

* *p < 0.1*.

*Refugees that migrated to Sweden between 1990 and 1993, at the ages of 25–55*.

Most of the control variables have the expected effects on the tendency to migrate internally within Sweden. Refugee men that are younger and were not employed in the previous year are more likely to migrate within Sweden. In addition, refugees that are not married tend more to migrate to both types of labor markets. Finally, there are significant ethnic differences in the internal migration patterns.

Some of the impacts of the labor market characteristics in the previous year on the probability of refugees' internal migration in Sweden are worth mentioning. First, as expected, high levels of unemployment in the labor market to which an individual was initially assigned encourage internal migration. Second, the structure of opportunities in the labor market in the year prior to migration has an opposite effect on each type of internal migration. An initial market with a wider structure of opportunities is associated with lower levels of internal migration to markets with similar (wider) structures of opportunities, and the opposite is true when starting in low structures of opportunities. These findings are probably, at least in part, the results of ceiling and floor effects.

Finally, and as expected, labor market ethnic concentration has the same effect on the two indices. Higher levels of ethnic concentration in the labor markets in which refugees were initially placed are found to be associated with lower chances of moving to other labor markets, regardless of the markets' structure of opportunities.

The results presented to this point confirm our hypotheses, namely that (1) refugees with higher education levels are more likely to migrate to wider-opportunities labor markets in which they benefit from their relatively high skills; and (2) refugees with lower education levels are more likely to migrate to narrower-opportunities markets in which they benefit more from their relatively low skills. The remaining question is whether those refugees who do migrate are indeed compensated for their move. This question is examined next.

### The Economic Consequences of Internal Migration

As noted above, economic theory holds that individuals who choose to move are expected to increase their employment probabilities and earnings. In order to try to assess the net effects of the internal migration decision, we track the refugees' income, before and after that migration takes place and then compare their income trajectories over time. This is done by using OLS fixed-effect models.

Table 3, shows estimates derived from individual fixed-effect earnings regression models—separately for refugee men and women. These models assess whether internal migration within Sweden is associated with positive effects on refugees' earnings and whether these positive effects can be interpreted in causal terms. The table displays the four parameters of interest: the first three represent the premiums gained (or lost) by refugees following their internal migration, while the fourth represents the self-selection effect on earnings (results of the full models are available upon request). First, it is possible that each type of internal migration has a constant effect on earnings subsequent to internal migration. Two parameters capture this effect, represented by “*move to more”* and “*move to less”* opportunities-structure markets. In addition, the parameter “*years after”* the internal move captures any additional annual income growth occurring after internal migration, where a positive coefficient indicates an annual premium after the internal move. Finally, a positive coefficient of “*years before”* the internal move indicates earnings growth enjoyed by individuals who eventually migrate occurring prior to the internal migration itself, implying that the individuals who decide to move are positively selected.

**Table 3:** OLS individual fixed-effect estimates of the effects of internal migration on (ln) income from work and self-employment of refugees^(1)^.

	**Yugoslavia**	**Croatia and Slovenia**	**Bosnia-Herzegovina**	**Macedonia**	**Somalia**	**Lebanon**	**Iraq**	**Iran**
**Variables**	**Men**							
Moved to more (constant premium)	−1.120^***^	−0.234	−0.443^***^	−0.847	−0.070	−0.449^*^	−0.875^***^	−0.332^*^
	(0.225)	(0.791)	(0.121)	(1.128)	(0.228)	(0.251)	(0.155)	(0.169)
Move to less (constant premium)	−0.196	−0.788	0.834^***^	3.208^*^	−0.443	0.530	0.684^**^	0.054
	(0.288)	(1.187)	(0.177)	(1.687)	(0.386)	(0.378)	(0.266)	(0.304)
Years after (slope premium)	0.588^***^	0.922^***^	1.197^***^	0.616^***^	0.545^***^	0.548^***^	0.721^***^	0.792^***^
	(0.020)	(0.074)	(0.021)	(0.086)	(0.033)	(0.026)	(0.016)	(0.018)
Years before (selection)	0.263^***^	0.213	−0.045^**^	−0.098	0.003	0.034	0.022	0.019
	(0.041)	(0.148)	(0.022)	(0.213)	(0.048)	(0.048)	(0.030)	(0.034)
Observations (individual ^*^ year)	18,449	1,330	39,894	833	8,650	11,725	28,640	23,562
*Individuals*	2,457	176	5,353	108	1,178	1,580	3,739	3,120
*R*-squared	0.191	0.343	0.423	0.219	0.088	0.079	0.154	0.168
rho	0.496	0.538	0.571	0.518	0.371	0.431	0.397	0.438
sigma_e	4.081	3.942	3.849	3.862	3.974	4.067	3.958	4.045
sigma_u	4.045	4.257	4.436	4.003	3.049	3.537	3.213	3.568
	**Women**							
Moved to more (constant premium)	−0.466^**^	−1.397^*^	−0.465^***^	−0.711	0.139	−0.689^***^	−0.241	−0.676^***^
	(0.224)	(0.840)	(0.121)	(1.375)	(0.236)	(0.249)	(0.209)	(0.175)
Move to less (constant premium)	0.298	−0.826	0.739^***^	0.501	0.368	−0.551	0.364	−0.036
	(0.320)	(1.384)	(0.184)	(1.789)	(0.428)	(0.403)	(0.349)	(0.319)
Years after (slope premium)	0.657^***^	0.922^***^	1.116^***^	0.500^***^	0.399^***^	0.376^***^	0.599^***^	0.692^***^
	(0.020)	(0.078)	(0.021)	(0.117)	(0.027)	(0.023)	(0.018)	(0.016)
Years before (selection)	0.128^***^	0.383^***^	−0.043^*^	0.506^**^	−0.058	−0.036	−0.032	0.083^**^
	(0.041)	(0.145)	(0.022)	(0.246)	(0.047)	(0.046)	(0.039)	(0.034)
Observations (individual ^*^ year)	16,170	1,343	37,457	494	5,101	7,347	15,317	21,396
*Individuals*	2,146	178	4,989	64	668	981	1,971	2,798
*R*-squared	0.220	0.308	0.401	0.215	0.095	0.073	0.147	0.166
rho	0.520	0.563	0.540	0.560	0.466	0.420	0.393	0.400
sigma_e	3.800	4.000	3.709	3.955	2.823	2.947	3.419	3.670
sigma_u	3.958	4.536	4.020	4.466	2.635	2.506	2.751	2.998

(1) Other variables that are included in the model are YSM, education, age, married, and labor market-level variables (see “Variables” section). Standard errors in parentheses;

*** p < 0.01,

** p < 0.05,

* *p < 0.1*.

As can be seen from Table 3, all immigrant men and women have a higher total earnings growth after internal migration as compared to their own pre-move earnings and the earnings of non-movers (stayers). This positive and significant total effect is substantial.

For example, a look at immigrant men from Yugoslavia shows that, first, they are positively selected into internal migration within Sweden: those who eventually migrate have a steeper earnings growth by about 30 percent (*b* = 0.263) even before moving, as compared to those immigrant men from Yugoslavia who stay. Second, while the migration itself distorts the earnings growth they had been experiencing prior to their move, their earnings after migrating are substantially higher. Those who moved to a market with a wider opportunities structure experience a reduction of about 206 percent (*b* = −1.12) in their earnings following that move (a shift effect). However, since their earnings growth is constantly increasing by about 80 percent per year after migration (*b* = 0.588), their earnings growth is expected to again be positive in <3 years after their move. Those who move to a narrower opportunities market do even better. They suffer a much smaller shift penalty, but enjoy the same annual rate of increase in their earnings as their counterparts who chose the markets with wider opportunities structures.

Clearly, the effects of internal migration on refugees' earnings growth are substantial. All movers, whether to wider- or narrower-opportunities markets, much accelerated (on average) the rate of their earnings growth. Several factors may explain these high premiums to internal migration. First, as indicated by the positive self-selection coefficients, the movers possess higher-than-average (mainly unobserved) earnings determinants. They belong to a relatively small group (about one-third of all refugees) that is, probably, extremely selective. Second, we estimate their rising earnings slope (“slope premium”) based on the first 7 years after their move. Usually, the steepest rise in such premiums occurs during the initial period right after the move. Therefore, our estimated slope premiums are probably overestimated when the entire working lifetime of the refugees is considered. Finally, many of the movers did not work prior to their move (see Table 2). Consequently, the rise in the movers' earnings is expected to be higher (on average) than that among stayers.

Based on Table 3, we can conclude that most of those refugees, both men and women, that move to markets with wider structures of opportunities experience a negative shift in their earnings immediately after their migration. A few groups moving in a similar direction experience a positive shift in their earnings. When we look at the refugees that move to markets with fewer opportunities, the effects are reversed. Most of the male and one female origin groups enjoy a positive shift in their earnings following such a move.

But more importantly, all groups enjoy a very steep rise in the rate of their earnings growth in subsequent years that offsets the shift penalties (or adds to the positive impacts) and substantially improves their earnings after the move as compared to their earnings before (and relative to stayers) within a few years^16^.

## Summary and Conclusions

The purpose of the present study has been to examine a very important issue involved in refugees'-integration policies. We studied the extent to which the economic theory of self-selection of immigrants to destinations based on their abilities applies not only to economic immigrants across countries, but to internal migration of refugees as well. The paper evaluates first whether highly skilled refugees, similarly to international economic migrants, tend to migrate within their destination country to labor markets with broader opportunity structures, while less capable individuals choose markets that are more sheltered. Second, the paper evaluates the extent to which such migration decisions improve the economic standing of refugees.

To do so, we use what can be described as a natural experiment, focusing on a refugee cohort that came to Sweden during a period when the “*Whole-Sweden*” policy was in effect. This policy was designed to reduce the concentration of refugees, mainly in the large cities, by randomly deploying asylum seekers to almost all municipalities within Sweden. After being assigned to an initial location, these refugees were given a choice whether to stay in their assigned location or move to another place within Sweden. This allows us to examine refugees' self-selection patterns within Sweden and the effects of those patterns on refugees' subsequent economic assimilation.

The results support our research hypotheses. In line with our expectations, we find that refugees' education levels are related to major differences in their destination choices. Highly skilled refugees are more likely to migrate to labor markets with wide structures of opportunities relative to less-skilled ones. In addition, we find that internal migration among refugee men and women in Sweden, whether to a wider or narrower market, is associated with a steeper rise in their annual earnings, thus making the move a rational decision that is very beneficial economically. This suggests that even among refugees, internal migration decisions are based also on economic maximization considerations.

**Figure 1:**
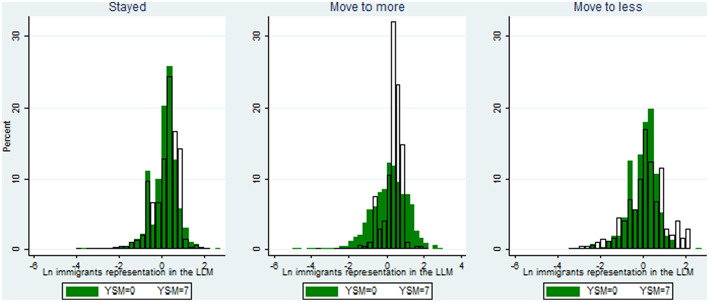
Levels of ethnic concentration at the first and at the seventh year after migration to Sweden—by type of internal migration.

However, as described in the theoretical section of this paper, there are also other motives for internal migration, among which the most important is the refugees' desire to live in an environment with a high representation of their own ethnic group. Clearly, such motives cannot be defined exclusively as “non-economic” because such a living environment allows refugees to create and strengthen their social networks and, consequently, to raise their employment and earnings opportunities. Indeed, some support for the mixed social-economic “networks motive” can be derived from the data. Figure 1 presents the levels of concentration at the first year and 7 years after immigration to Sweden by type of internal migration. An ethnic concentration value of 0.0 indicates a level of ethnic concentration in local labor markets that is similar to the national ethnic concentration level, while positive (negative) values indicate ethnic concentration in local labor markets that are higher (lower) than that at the national level. As can be seen, refugees that do not end up moving from their initial locations were placed, from the beginning, in labor markets with members of their own ethnic group (values above zero imply that the percentage of the ethnic group in the local labor market exceeds their share of the entire population). That is, most of them were initially placed in localities in which their own ethnic group was overrepresented (as can be seen by a more skewed distribution in which the peak exceeds the value of zero). Seven years after their initial placement, the levels of their labor market concentration are somewhat even higher—as shown by the high peak around the value 1.0 in year 7. This higher level of ethnic concentration could be a result of internal movements of refugees of their own ethnic groups to the labor markets in which the refugees were initially placed.

Contrary to this group, immigrants that end up moving to labor markets with wider structures of opportunities lived upon their arrival in localities with more symmetrical concentration distributions, with a high share of them living in labor markets with an underrepresentation of members of their own ethnic group. However, after 7 years in Sweden, most of them are found in labor markets with higher levels of representation of their own groups. This trend resulted in a distribution with an even higher peak around the value 1.0 (a value representing concentration) than that of immigrants that initially were placed in high-concentration cities and stayed there.

Finally, immigrants who end up moving to labor markets with narrower structures of opportunities also belong to a quite symmetrical initial distribution in terms of their ethnic concentration, with a high share of them located in labor markets with overrepresentation of their co-ethnic refugees as compared to immigrants that moved to labor markets with wider structures of opportunities. After 7 years, some of them move to labor markets with even higher levels of ethnic concentration, while some, however, move to less ethnically concentrated labor markets—a trend that is unique to this group.

The selectivity in the patterns of internal migration of refugees has implications for dispersal policies. The main purpose of the Swedish dispersal policy was to ease the burden of the refugee flows on the large cities by lowering the levels of ethnic concentration of refugees in those cities. However, we show that the concentration equilibrium created by such a policy is unstable. Refugees who end up moving to different labor markets were initially placed in localities where their own ethnic group had lower levels of representation, relative to the markets in which those that stay were initially placed. This suggests that stayers, as well as movers, might prefer localities with a high share of their own ethnic group. Such preferences might result, not only from possible economic gains associated with it, but also due to the ethnic goods of living near their co-ethnic members (e.g., social well-being, ethnic identity).

## Author Contributions

All authors listed have made a substantial, direct and intellectual contribution to the work, and approved it for publication.

### Conflict of Interest Statement

The authors declare that the research was conducted in the absence of any commercial or financial relationships that could be construed as a potential conflict of interest.

## Footnotes

^1^The above assumption was indeed refuted empirically. A significant correlation between individuals' skill levels and average skill levels in the region of residence at the age of 14 was found.

^2^Sometimes, a migration decision echoes a wish to simply change living environment (Vilhelmson and Thulin, 2016).

^3^Administrated by the unit of Human Geography at the University of Gothenburg. It comprises official register data provided by Statistics Sweden from the database Longitudinell Integrationsdatabas för Sjukförsäkrings- och Arbetsmarknadsstudier (LISA). It contains data on every individual, 16 years or older, registered in Sweden as of the 31st of December each year.

^4^Yugoslavia was founded after the 2nd World War, and was made up of six Balkan constituent federal units: Bosnia and Herzegovina, Croatia, Macedonia, Montenegro, Serbia, and Slovenia. The Yugoslav War led to the breakup of this federation by mid-1992, and only Serbia and Montenegro remained united (until 2006) under the name “Yugoslavia.”

^5^Individuals were omitted from our sample if they died or emigrated from Sweden during the 7-year period following their arrival.

^6^https://www.scb.se/hitta-statistik/statistik-efter-amne/arbetsmarknad/sysselsattning-forvarvsarbete-och-arbetstider/registerbaserad-arbetsmarknadsstatistik-rams/produktrelaterat/Fordjupad-information/lokala-arbetsmarknader-la/forteckning-over-lokala-arbetsmarknader/

^7^Because there were changes in the definitions of municipalities in Sweden over the years, we first harmonized the municipality coding to fit the 1990 definition and then modified the classification of the labor markets as defined by SCB in 1990. SCB's definition of labor markets is based on the factual patterns of work-related commuting within and between municipalities.

^8^A significant share of refugees does not have positive earnings in each one of the years studied. Therefore, we assign the value of '1' to these cases in order to be able to include them in the models (where their earnings are equal to ln(1) = 0).

^9^If an individual moves a second time, then the subsequent move is censored.

^10^Elementary education is defined as up to 9 years.

^11^In the fixed effects models we did not include a variable of years as it highly correlates with our variables that assess the selection to and the premium from internal migration. This is mainly due to the relatively small numbers of years of migration and the time span in which we are tracking these immigrants.

^12^This procedure might, of course, lower the magnitude of the standard errors associated with the estimated coefficients and, consequently, lead to Type-I statistical errors. However, this is not a real problem in studies such as ours in which the entire population is used.

^13^Note that some of these differences are a result of ceiling and floor effects. In order for individuals to be classified as movers to markets with wider opportunities structures, they need to start at a market with a narrower structure of opportunities, and vice versa.

^14^It should be noted that these figures are based on differences in means only. However, the shapes of the concentration distributions of the three groups are different, and we discuss the possible impacts of the internal migration decisions on those shapes in the final section of the results.

^15^In general, we include the entire population of the 1990–1993 refugee cohort in all our estimated models. Thus, we are not referring to statistical significance issues in describing our results. However, the results tables indicate significance levels for those who are interested in treating the parameters presented as if they are estimated statistics.

^16^In addition to the division of the refugees studied into countries of origin, we grouped the refugees into two groups of regions of origin: the first includes refugees from Europe, the second—refugees arriving to Sweden from Africa and the Middle East. The results of the estimated earnings model under this categorization are appreciably the same as presented in Table 3, and available upon request.
